# When to quit: Calibration of voluntary persistence and attempted suicide in depression

**DOI:** 10.1016/j.brat.2026.105148

**Published:** 2026-09-05

**Authors:** Aliona Tsypes, Joseph T. McGuire, Alexandre Y. Dombrovski

**Affiliations:** a Department of Psychiatry, University of Pittsburgh, Pittsburgh, PA, USA; b Department of Psychological and Brain Sciences, Boston University, USA

**Keywords:** Suicide, Depression, Decision-making, Willingness-to-wait task, Voluntary persistence calibration

## Abstract

**Background:**

During a suicidal crisis, people may repeatedly reconsider how to respond as distress continues and prospects for relief change. One hypothesis links suicidal behavior to a preference for immediate escape over future rewards, but delay discounting findings are mixed. These studies typically assess choices between sooner and later rewards, leaving unresolved how people decide whether to persist as time costs accumulate. We examined re-engagement after setbacks and calibration of persistence in depression and attempted suicide.

**Methods:**

Adults from a mid- to late-life depression cohort (*N* = 277; 87 attempters, 59 ideators, 63 depressed non-suicidal, 68 controls) completed the Willingness-to-Wait task, in which waiting up to 3 s maximized reward, whereas longer waits were counterproductive. Immediate quitting – taking the smaller reward without waiting – was modeled with multilevel logistic regression; quitting latency once waiting began with Cox mixed-effects models. Exploratory analyses examined attempt lethality and age of first attempt. Sensitivity analyses accounted for impulsivity, cognition, and personality.

**Results:**

All groups were more likely to quit immediately after losses than wins, but this difference was largest in attempters. Once a wait began, attempters and depressed non-suicidal participants overpersisted relative to controls. Earlier age of first attempt, but not attempt lethality, was linked with stronger loss-triggered quitting. Findings were robust to confounds.

**Conclusions:**

Depression was associated with maladaptive overpersistence, whereas a history of suicide attempts was additionally associated with loss-triggered disengagement. Dynamic tasks that separate re-engagement after failure from stopping decisions may help clarify decision processes relevant to suicidal behavior.

## 1. Introduction

What determines the moment someone attempts suicide? A suicidal crisis may unfold while a person is scrolling through distressing social media posts, mentally replaying an argument, walking down a busy street, or perhaps planning a suicide attempt. Such moments are often marked by ambivalence: a simultaneous pull toward escape from seemingly intolerable distress and toward survival, rescue, or connection. In this state, the consequences of acting or waiting can change rapidly. Acting quickly may promise immediate relief but also foreclose the possibility that distress will subside, that another person will intervene, or that a coping option will become available. Waiting may allow time for help-seeking, reconsideration, or rescue, but it can also feel increasingly costly as distress continues and as time-sensitive sources of support or safety become unavailable. Decision-making in this context is dynamic, uncertain, and cognitively demanding.

One influential hypothesis holds that suicidal behavior reflects a myopic preference for immediate over delayed outcomes, such that escape from an aversive state is chosen at the expense of future rewards. Delay discounting refers to how much people devalue outcomes as the delay before receiving them increases, typically measured by choices between smaller-sooner and larger-later rewards. Evidence from delay discounting studies is mixed. While some reported steeper delay discounting in suicidal behavior (Dombrovski et al., 2011; Dougherty et al., 2009; Liu et al., 2012; Mathias et al., 2011), others found no evidence of myopic preferences (e.g., Bridge et al., 2015; Millner et al., 2020; Tsypes et al., 2022). These inconsistencies likely reflect, in part, clinical heterogeneity across samples and subtypes of suicidal behavior, including differences in age, diagnosis, attempt lethality, planning, and substance use. They may also reflect methodological factors. For example, publication bias can inflate apparent effects, and two-stage discounting analyses often estimate a single discount rate for each person before comparing groups. When participants make inconsistent choices, these estimates can be imprecise, and the uncertainty in person-level estimates is not carried forward to the group-level analysis. As a result, choice inconsistency may be confounded with discount rates (Amlung et al., 2019; Tsypes et al., 2022). Crucially, however, delay discounting paradigms tend to rely on discrete choices between descriptively presented, often hypothetical, prospects that do not require people to experience a temporally extended period of waiting while re-evaluating their decision. Yet it is precisely this type of experience that places critical demands on decision-making in a suicidal crisis.

One process not captured in previous studies of suicidal behavior is reconsidering an ongoing course of action as the chances of success shift and opportunity cost accumulates. Behavioral economists frame this problem as the calibration of voluntary persistence under uncertainty: deciding how long to continue waiting given elapsed time and recent outcomes. For example, the Willingness-to-Wait (WTW) paradigm (McGuire & Kable, 2012, 2015) asks participants to decide, on each trial, whether to continue waiting for a larger temporally uncertain reward or to quit and move on. Prior WTW studies show that people can use the timing statistics of reward delivery to calibrate persistence: when continued waiting makes reward more likely or more imminent, they wait longer; when elapsed time signals that the remaining delay is likely to be long, they learn to limit waiting and start a new trial instead (e.g., Fung et al., 2017; Lang et al., 2022; Lempert et al., 2018). Thus, WTW provides a way to study how people learn from experience when persistence is useful and when quitting is the better policy.

When a smaller immediate reward and a larger delayed reward are both available, the decision can be approached as a set of two distinct *start* and *stop* decisions. The first is whether to *start* waiting for the larger delayed reward or opt for a smaller immediate alternative at the beginning of a trial. This starting decision depends on the outcome of the preceding trial: after a rewarded trial, starting another wait repeats a recently successful action, whereas after an unrewarded trial, taking the small immediate reward abandons it. This outcome-dependence reflects the well-established win-stay/lose-shift tendency in feedback-guided choice (e.g., Frank et al., 2004; Worthy et al., 2013). Starting a new wait after a loss is thus a form of re-engagement—persisting with a potentially rewarding action despite a recent failure—whereas quitting immediately forgoes that opportunity. Evidence from several paradigms suggests that this pattern is disrupted in individuals at risk for suicide. In a probabilistic reversal learning study, suicide attempters displayed excessive shifting after losses even when prior history favored staying the course (Dombrovski et al., 2010). In an escape decision task, suicidal participants displayed a heightened bias for active escape and performed worse when relief required inaction, indicating reduced preference for passive waiting when an immediate action is available (Millner et al., 2019). Our recent study of exploration-exploitation similarly found disrupted outcome-contingent shifting in suicide attempters across two samples with borderline personality and late-life depression (Tsypes et al., 2024).

A loss-triggered tendency to disengage is not inherently maladaptive. Shifting away after an unrewarded outcome is sensible when recent outcomes predict upcoming ones and, when waiting carries an opportunity cost, acting sooner frees time for other opportunities. Indeed, normative analyses of decisions under time cost show that it can be optimal to require progressively less evidence to act as time passes (Drugowitsch et al., 2012; Tajima et al., 2016). However, in the WTW task, successive trials are independent, and a brief wait would have secured the reward on most trials. Thus, quitting immediately after a single unrewarded trial forgoes reward that brief persistence would likely have delivered. We therefore expected suicide attempters to exhibit an exaggerated, loss-triggered form of this tendency: *reduced re-engagement after failure*.

The second decision is when to *stop* the wait that is already in progress, once continued persistence no longer maximizes reward. This decision requires dynamically updating the assessment of whether the larger delayed reward remains worth the opportunity cost of continued waiting. Suicide attempters exhibit impaired reinforcement learning and noisier discrimination between closely valued alternatives (Dombrovski et al., 2010, 2019), paralleled by attenuated ventromedial prefrontal expected value signals (Brown et al., 2020; Dombrovski et al., 2013). Accordingly, during delay discounting, attempters’ valuation of reward appears to be noisy rather than myopic (Tsypes et al., 2022). These observations suggest *impaired dynamic re-evaluation of whether continued persistence remains worthwhile*.

To identify deficits specific to suicidal behavior rather than depression or suicidal ideation, we recruited four groups of participants: depressed people with a verified history of suicide attempts, depressed people with suicidal ideation but no attempts, depressed people without suicidal thoughts or behavior, and non-depressed healthy controls. We sampled people with mid-life and particularly late-life depression because suicide attempts in later life are often medically serious, making this cohort clinically informative for studying suicidal behavior that closely resembles fatal attempts (Conwell et al., 1998; De Leo et al., 2001; Dombrovski et al., 2008). Clinician-verified histories and comprehensive clinical and cognitive characterization supported thorough sensitivity analyses to rule out alternative explanations. The decision processes we examine are most relevant to suicidal crises that involve some deliberation, since suicide attempts that occur with little planning or without acknowledged suicidal ideation may engage partly different processes (Wastler et al., 2022; Wyder & De Leo, 2007).

We tested two primary hypotheses. First, we hypothesized that suicide attempters would show reduced re-engagement after failure, operationalized as stronger loss-triggered immediate quitting at the start of the next trial, and tested with trial-level mixed-effects logistic models. Second, we hypothesized that suicide attempters would show poorer calibration of stopping once a wait had begun, operationalized as suboptimal quitting latency on non-immediate-quit trials and tested with Cox mixed-effects models. Because the reward-maximizing policy was to quit after 3 s if the larger reward had not yet arrived, longer quitting latencies indexed overpersistence. Finally, prior work indicates that high relative to low attempt lethality is differentially implicated in learning abnormalities, altered exploration, and reward valuation consistency (e.g., Dombrovski et al., 2019; Tsypes et al., 2022, 2024) and that early- and late-onset attempts show distinct profiles of affective reactivity, cognitive rigidity, and decision outcomes in late life (e.g., Perry et al., 2021; Szanto et al., 2024; Szücs et al., 2020). In the present study, we therefore treated medical lethality and age of first attempt as clinically important dimensions and planned exploratory tests of whether WTW indices of persistence calibration varied along these dimensions within the attempter group.

## 2. Methods

### 2.1. Participants and recruitment

Participants were drawn from a longitudinal study of suicidal behavior in mid- and late-life depression conducted in Pittsburgh, Pennsylvania. This sample included 277 participants aged 42–92 (*M*age = 63.6, *SD* = 9.06; 49.50% female) and comprised four groups: 87 suicide attempters with depression (attempters), 59 suicide ideators with depression (ideators), 63 nonsuicidal participants with depression (depressed non-suicidal), and 68 healthy controls (controls). The procedures were approved by the University of Pittsburgh Institutional Review Board. All participants provided written informed consent. Recruitment efforts were conducted through a psychogeriatric inpatient unit, a late-life depression clinic, primary care, and community advertisements.

### 2.2. Diagnostic assessment and group classification

Diagnoses were established with the Structured Clinical Interview for DSM-IV Axis I Disorders (SCID-I; First et al., 1996). A suicide attempt was defined as a potentially self-injurious act with a nonfatal outcome for which there was explicit or implicit evidence of intent to die (O’Carroll et al., 1996). The presence of suicide attempt history was verified by a psychiatrist who used all available information: participant’s report, medical records, and collateral information from the treatment team, family, and friends. Significant discrepancies between these sources led to exclusion from the study. The Beck Lethality Scale (Beck et al., 1975) was used to determine the medical seriousness of suicide attempts. If a participant had a history of multiple attempts, the data from their most lethal attempt were used. Attempts were classified as high-lethality if the Beck Lethality Scale score was 4 or greater and as low-lethality otherwise. Early- versus late-onset attempters were defined by whether the first suicide attempt occurred before versus at or after age 50. The Ideators group had suicidal ideation with a specific plan but no suicide attempt history. The depressed non-suicidal group included participants with no lifetime history of any suicidal or nonsuicidal self-harming thoughts or behaviors. At study enrollment, all three depressed groups had a SCID-I lifetime diagnosis of unipolar nonpsychotic depressive disorder and a score of 14 or higher on the Hamilton Rating Scale for Depression (HRSD-17; Hamilton, 1960). Controls had no lifetime history of SCID-I psychiatric diagnoses. Key demographic, clinical, cognitive, and personality characteristics are presented in Table 1.

### 2.3. Clinical, cognitive, and personality measures

Depression severity was assessed with the 17-item Hamilton Rating Scale for Depression (HRSD-17; Hamilton, 1960). Table 1 reports the baseline HRSD-16 score, calculated by excluding the suicide item from the HRSD-17 total. Estimated verbal IQ was assessed with the Wechsler Test of Adult Reading (WTAR; Holdnack, 2001). Executive functioning was assessed with the Executive Interview (EXIT25; Royall et al., 1992), with higher scores indicating worse executive function. Impulsivity was assessed with the Barratt Impulsiveness Scale (BIS-11; Patton et al., 1995) and the UPPS-P Impulsive Behavior Scale (Cyders et al., 2007; Whiteside & Lynam, 2001). Big Five personality traits were assessed with the NEO Five-Factor Inventory (NEO-FFI; Costa & McCrae, 1992). These measures were included to characterize group differences and to support sensitivity analyses evaluating whether WTW effects were explained by cognitive functioning, impulsivity, or personality.

### 2.4. Willingness to wait paradigm (Fig. 1)

**Fig. 1.**
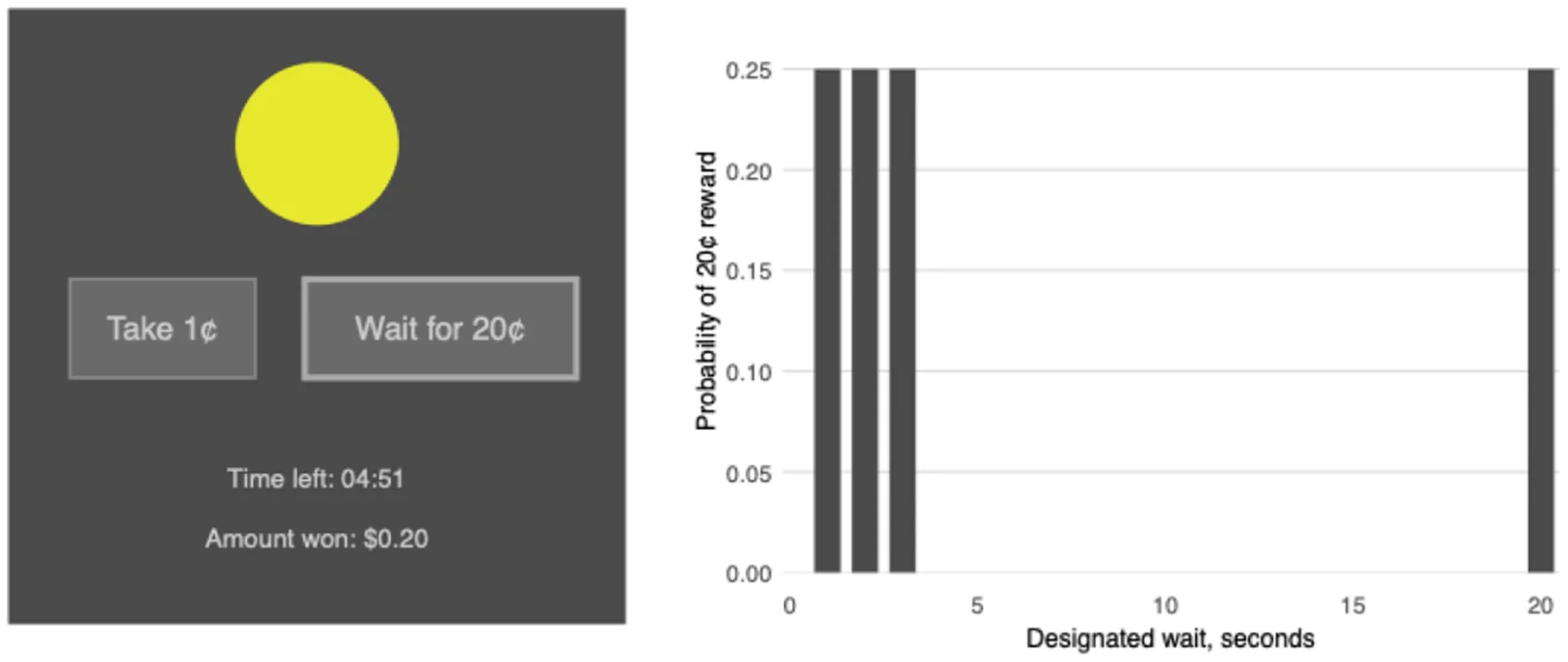
Willingness to Wait (WTW) Paradigm. Left: On each trial, participants could leave the mouse cursor in the right-hand box to wait for the larger reward or could move the cursor to the left-hand box at any time to take the smaller immediate reward and end the trial. Right: The large reward on each trial was scheduled to arrive at a delay of 1, 2, 3, or 20 s, with equal probability. The theoretical reward-maximizing strategy was to wait 3 s and then give up and take the small reward if the large reward had not yet arrived.

Participants completed the Willingness to Wait voluntary temporal persistence task (McGuire & Kable, 2012), involving repeated opportunities to wait for temporally uncertain rewards. The task lasted 5 min, during which participants completed as many trials as time allowed. Trials were separated by a 2-s inter-trial interval. On each trial, participants could leave the mouse cursor in the right-hand box to continue waiting for the larger reward or move the cursor to the left-hand box at any time to collect the smaller immediate reward and end the trial (see Fig. 1). The larger reward was scheduled to arrive after a delay of 1, 2, 3, or 20 s with equal probability. Participants were not told the reward timing distribution or the optimal giving-up threshold. A trial was coded as rewarded, or a prior-trial win, if the participant waited until the larger reward arrived. A trial was coded as unrewarded, or a prior-trial loss, if the participant quit before the larger reward arrived, regardless of the scheduled delay. Immediate quitting was defined as quitting at the start of a trial without initiating a wait. Because all short-delay rewards could be obtained by waiting through 3 s, whereas waiting beyond 3 s committed time to the rare 20-s reward, the reward-maximizing strategy was to wait up to 3 s and then quit if the larger reward had not arrived.

### 2.5. Statistical analyses

Analyses focused on two behavioral outcomes corresponding to the two decisions described in the Introduction: immediate quitting at the start of a trial and quitting latency once waiting had begun. Analyses were conducted in R version 4.3.1 (R Core Team, 2023) using lme4 for logistic mixed-effects models (Bates et al., 2015), survival for Kaplan-Meier/log-rank analyses (Therneau, 2023; Therneau & Grambsch, 2000), and coxme for Cox mixed-effects models (Therneau, 2024).

Immediate quitting was analyzed with mixed-effects logistic regression. The dependent variable was whether the participant quit immediately at the start of the trial. Fixed effects were group, prior-trial outcome (rewarded vs. unrewarded), and the group-by-prior-trial outcome interaction. Participants were included as random intercepts. The attempter group was the reference group in the primary model, allowing other groups to be compared directly with attempters. Trials following immediate quits were excluded to reduce confounding by choice autocorrelation, because an immediate quit provides no opportunity to observe whether the larger reward would have arrived.

Quitting latency was analyzed on trials that were not immediately abandoned. Because trials in which participants waited until reward receipt yielded right-censored observations of willingness to wait, we first used Kaplan-Meier survival curves and the log-rank test to compare quitting latencies across groups. We then used Cox mixed-effects models to predict quitting latency from group, prior-trial outcome, and the group-by-prior-trial outcome interaction, with participant included as a random intercept. In the Cox models, negative coefficients indicate lower hazard of quitting and therefore longer persistence. Controls were the reference group in the primary Cox model. To describe the practical magnitude of overpersistence, we compared waiting behavior with the 3-s reward-maximizing threshold. We summarized the typical timing of delayed quit decisions and the percentage occurring after 3 s. We also calculated each participant’s average excess waiting across all non-immediate quit trials, defined as the number of seconds beyond 3 s on each trial, with trials ending by 3 s contributing zero.

Exploratory analyses examined heterogeneity within suicide attempters by medical lethality and age of first attempt. These models paralleled the primary immediate-quitting and quitting-latency models, replacing the broader attempter group with the relevant attempter subgroups.

To evaluate robustness to demographic and cognitive differences between groups, we estimated covariate-adjusted versions of the two primary models including age, education, WTAR-estimated IQ, and EXIT25 executive functioning as covariates. These covariates were entered as main effects to preserve a parsimonious model. Additional sensitivity analyses examined whether effects were robust to impulsivity and personality measures and to exclusion of participants who never moved the cursor during the task.

### 2.6. Power analysis

Because our sample size was determined by funding and recruitment constraints for this thoroughly characterized, difficult-to-recruit clinical cohort, we evaluated the sensitivity of the current design using simulations matched to the primary mixed-effects models and repeated-trial structure (Green & MacLeod, 2016; Hoenig & Heisey, 2001; Lakens, 2022). This approach answers the relevant power question: What effect magnitude could the current sample and primary models detect with 80% probability? At each candidate effect magnitude, 1000 datasets were simulated and reanalyzed using a two-sided α of 0.05. The 80% minimum detectable effect was defined as the effect magnitude detected in 80% of simulations. Sensitivity was evaluated for the smallest focal immediate-quitting interaction and the attempter-versus-control quitting-latency effect.

## 3. Results

### 3.1. Main analyses

#### 3.1.1. Manipulation checks (Table 2 panel A, Supplemental Fig. 1)

As expected, participants were much less likely to quit immediately at the start of a trial after a win, defined as having received the large reward on the previous trial, than after a loss (β = −4.08, *p* < .0001), defined as having quit the previous trial before the large reward arrived. The pooled Kaplan-Meier analysis showed longer persistence after wins than losses. Participants also learned to quit faster over time if the early reward had not arrived after 3 s.

#### 3.1.2. Logistic mixed-effects model for immediate quitting (Table 2 panel A, Fig. 2)

Participants could quit at the start of a trial by taking the smaller reward without waiting for the larger one. We examined group differences in this behavior with a logistic multilevel model predicting immediate quits as a function of group (controls, attempters, depressed non-suicidal, ideators), prior trial win or loss, and their interaction. In this model, attempters served as the reference group. These analyses excluded the trials that followed immediate quits to avoid confounding by choice autocorrelation (runs of immediate quits).

**Fig. 2.**
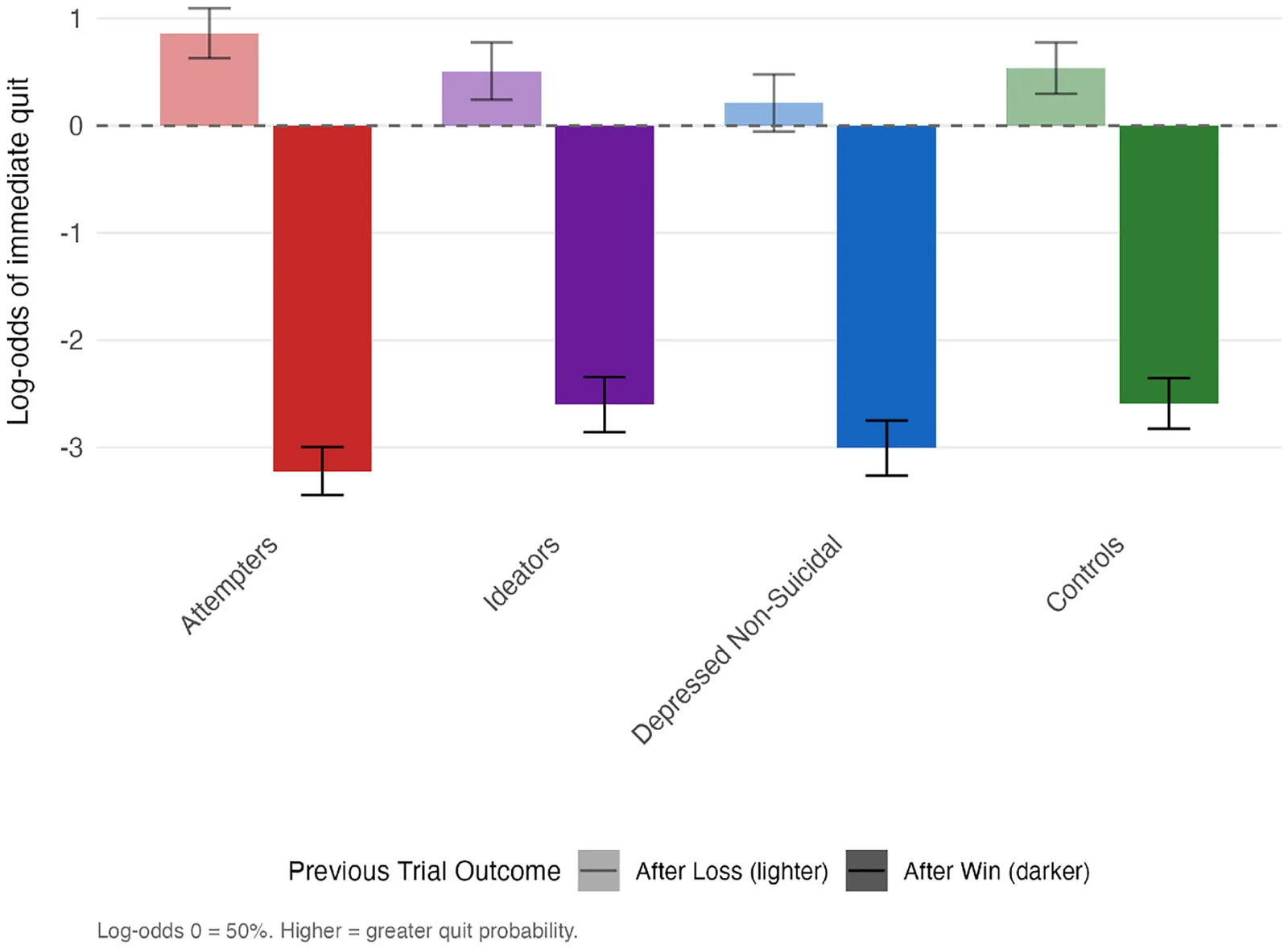
Immediate Quitting by Group and Prior Trial Outcome. Bars show model-estimated log-odds. The difference between losses and wins was larger in attempters than in the comparison groups. Error bars represent ±1 standard error.

Participants were less likely to immediately quit following a prior-trial win compared to a loss (β = −4.08, *p* < .001), whereas group membership had no main effect on immediate quits (χ^2^(3) = 3.45, *p* = .33). We observed, however, an interaction between group and prior trial outcome (χ^2^(3) = 20.70, *p* < .001). Follow-up analyses revealed that the effect of prior-trial outcome on immediate quitting was significantly stronger in suicide attempters than in controls (β = 0.96, *p* < .0001), depressed non-suicidal participants (β = 0.87, *p* = .0008), and ideators (β = 0.97, *p* = .0001). As illustrated in Fig. 2, attempters showed the largest increase in immediate quitting after losses relative to wins.

#### 3.1.3. Survival analysis of quitting behavior across groups on non-immediate quit trials (Table 2 panel B, Fig. 3)

Among the 2420 delayed-quit decisions, the median quitting latency was 4.52 s, or 1.52 s beyond the 3-s reward-maximizing threshold. Overall, 60.4% of these decisions occurred after 3 s. At the participant level, average excess waiting beyond 3 s per non-immediate-quit trial was 2.79 s (*SD* = 1.62) in attempters, 2.58 s (*SD* = 1.53) in depressed non-suicidal participants, 2.58 s (*SD* = 1.59) in ideators, and 2.20 s (*SD* = 1.44) in controls. The percentage of delayed-quit decisions occurring after 3 s was 65.6% in attempters, 62.2% in depressed non-suicidal participants, 53.8% in ideators, and 59.7% in controls.

**Fig. 3.**
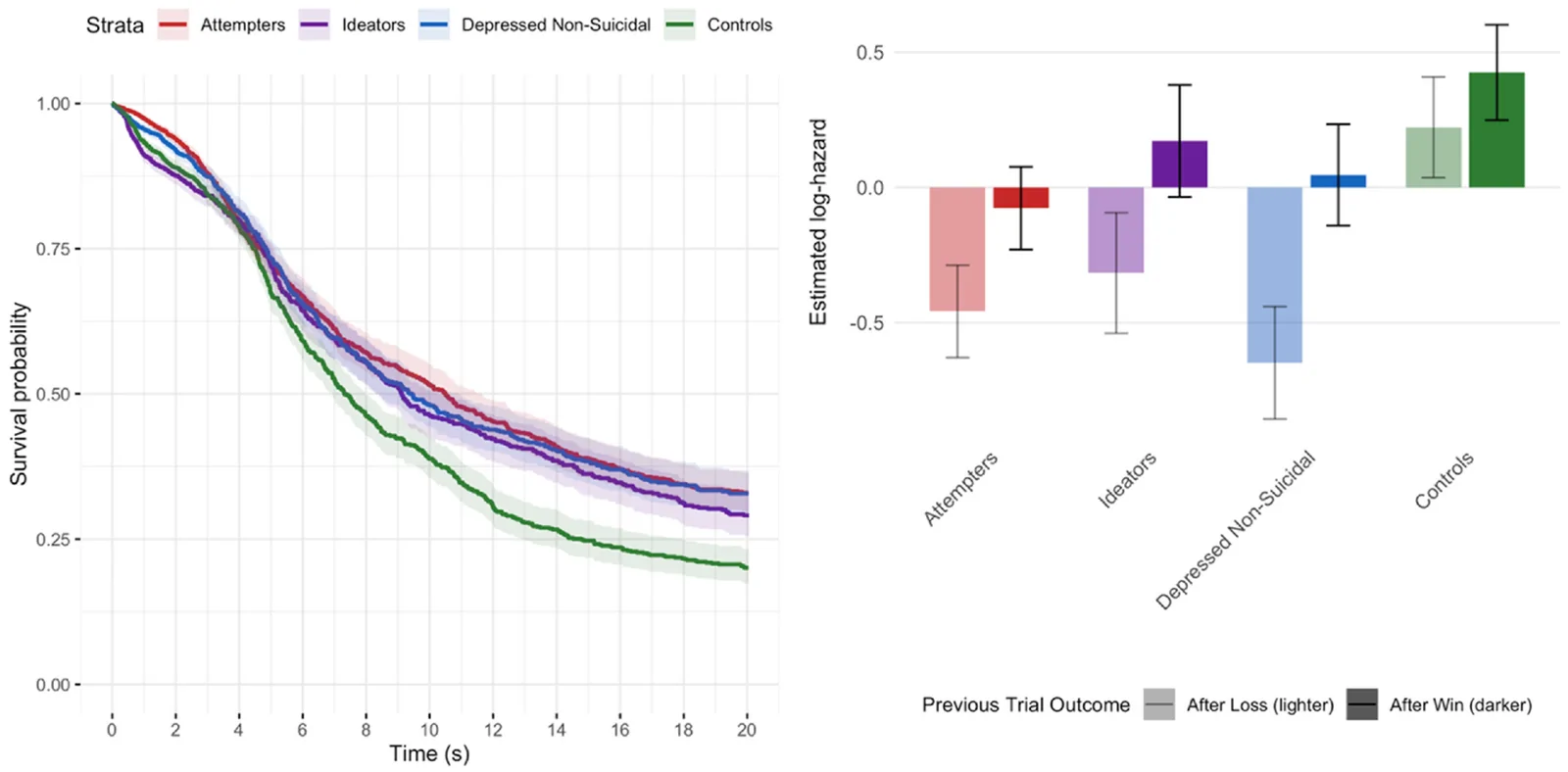
Persistence Once Waiting Began. Left: Kaplan-Meier survival curves by group, pooled across prior-trial outcomes. Shaded bands indicate 95% confidence intervals. Right: Cox mixed-effects model estimates of log quitting hazard by group and prior-trial outcome. Higher values indicate faster quitting. Error bars represent ±1 standard error.

To examine group differences in persistence against this backdrop, we first conducted a Kaplan-Meier (KM) survival analysis to compare quitting latencies across groups (controls, attempters, depressed non-suicidal, and ideators) and prior trial outcome (win or loss). Because some participants persisted throughout the trial without quitting, leading to right-censored data, the log-rank test was chosen to handle these cases appropriately. The log-rank test revealed significant differences in quitting latency between groups (χ^2^(3) = 59.6, *p* < .0001), indicating that group membership influenced persistence behavior (Fig. 3).

We evaluated these group differences further with a Cox multilevel model predicting persistence based on group, win or loss on the prior trial, and their interaction. This approach also ascertained that any apparent group differences were not explained by random inter-individual variability in persistence, reflected in person-level random intercepts. Both group membership (χ^2^(3) = 8.76, *p* = .03) and prior trial outcome (χ^2^(1) = 4.93, *p* = .03) predicted quitting behavior. Compared to controls, attempters (β = −0.68, *p* = .02) and depressed non-suicidal participants (β = −0.87, *p* = .006) exhibited significantly greater persistence. Ideators showed an intermediate level of persistence, not differing significantly from controls (β = −0.54, *p* = .11).

Groups also differed in how prior outcomes influenced subsequent persistence (χ^2^(3) = 11.48, *p* = .009). Relative to controls, depressed non-suicidal participants (β = 0.49, *p* = .001) and ideators (β = 0.28, *p* = .04) but not attempters (β = 0.18, *p* = .19) displayed a larger reduction in persistence following wins versus losses.

### 3.2. Exploratory analyses: Parsing heterogeneity of suicidal behavior (Supplemental Table S1–S4)

To understand how calibration of persistence is related to the characteristics of real-life suicidal behavior, we examined the effects of attempt lethality and age of onset. We found no distinct effects of high versus low attempt lethality (Supplemental Table S1 and S2). However, early-onset attempters showed stronger loss-triggered immediate quitting (Supplemental Table S3) and greater persistence than controls (Supplemental Table S4). Specifically, compared to early-onset attempters, controls (β = 2.22, *p* < .001), late-onset attempters (β = 1.90, *p* < .001), depressed non-suicidal (β = 2.13, *p* < .001), and ideators (β = 2.23, *p* < .001) showed less pronounced loss-triggered immediate quitting. Further, compared to controls, early-onset attempters (β = −0.93, *p* = .01) and depressed non-suicidal participants (β = −0.87, *p* = .006) exhibited significantly greater persistence, as indicated by longer quitting latencies.

### 3.3. Sensitivity analyses (Supplemental Table S5–S16)

The covariate-adjusted primary models included age, education, WTAR-estimated IQ, and EXIT25 executive functioning. Because of the missing WTAR/EXIT covariate data, the adjusted immediate-quitting model included 257 participants and 8468 trials, and the adjusted Cox model included 254 participants and 8382 trials. Within these complete-case samples, the key coefficients changed little after adjustment for covariates. In the immediate-quitting model, all three group-by-prior-outcome contrasts comparing attempters with the other groups remained significant: controls versus attempters, β = 0.97, odds ratio (*OR*) = 2.65, *p* < .001; depressed non-suicidal participants versus attempters, β = 0.96, *OR* = 2.62, *p* < .001; and ideators versus attempters, β = 0.96, *OR* = 2.60, *p* < .001. In the Cox model, following an unrewarded prior trial, quitting hazards remained lower than among controls for attempters, hazard ratio (*HR*) = 0.48, *p* = .010, and depressed non-suicidal participants, *HR* = 0.35, *p* < .001. In the complete-case sample, the ideator-versus-control estimate was near the significance threshold before adjustment, *HR* = 0.52, *p* = .053, and significant after adjustment, *HR* = 0.50, *p* = .030. Other Cox estimates remained similar in magnitude.

Further robustness analyses are summarized in Supplemental Table S5–S16. Excluding the 14 participants who never moved the cursor reproduced the primary pattern: attempter-specific immediate-quitting interactions remained significant, all *p* ≤ 0.0009, and attempters and depressed non-suicidal participants continued to show longer quitting latencies than controls, *p* = .023 and *p* = .007, respectively. Trait-screening models showed that several individual-difference variables moderated prior-outcome effects. In full models including these significant trait × prior-outcome terms, the central loss-triggered immediate-quitting result was not explained by any single trait: attempter-versus-control and attempter-versus-ideator interactions remained significant across the full trait-adjusted logistic models, although the attempter-versus-depressed non-suicidal comparison was attenuated in several personality-adjusted models. For quitting latency, depressed non-suicidal participants remained more persistent than controls across trait-adjusted Cox models, whereas the attempter effect was retained in the BIS Attentional model but attenuated to trend level in models including NEO-FFI Agreeableness and BIS Non-Planning. Thus, the sensitivity analyses supported the same directional interpretation while clarifying which effects were most robust when modeled alongside correlated individual-difference moderators.

### 3.4. Power analysis

Simulation-based sensitivity analyses indicated an 80% minimum detectable *OR* of 2.12 for the smallest focal immediate-quitting interaction and an 80% minimum detectable *HR* of 0.46 for the attempter-versus-control quitting-latency comparison. The observed immediate-quitting interaction (*OR* = 2.37) exceeded its sensitivity boundary. The observed latency estimate (*HR* = 0.51) was close to its boundary and was statistically significant, *p* = .018. The covariate-adjusted estimate was similar, *HR* = 0.48, *p* = .010.

## 4. Discussion

Whereas previous studies of temporal preferences in suicidal behavior generally relied on descriptions of options, we examined whether depressed people who attempted suicide were able to calibrate their persistence based on unfolding experience. We found, first, that suicide attempters were especially prone to quit immediately on the very next trial after a loss, a tendency not seen after wins and more pronounced than in controls, depressed non-suicidal people, or ideators. Second, once a wait began, attempters and depressed non-suicidal participants overpersisted relative to controls. These patterns were robust to potential confounds.

Our most striking finding was the propensity of suicide attempters to quit immediately after unrewarded trials, even though a brief wait would maximize rewards. This reduced re-engagement after losses is consistent with prior findings of overweighting of the most recent punishment in probabilistic reversal learning (Dombrovski et al., 2010), a bias toward active escape over more advantageous inaction (Millner et al., 2019), and inefficient outcome-contingent behavioral adjustment during exploration-exploitation decision-making (Tsypes et al., 2024). That this effect distinguished attempters from ideators, even though ideators here had suicidal ideation with a specific plan, speaks to the design’s central aim of isolating correlates of suicidal action rather than of suicidal thought. It suggests that the influence of a recent negative outcome on engagement with the next opportunity is more closely tied to a history of attempts than to planful ideation. Notably, this separation emerged for loss-triggered quitting but not for stopping calibration, on which ideators were intermediate, a dissociation we return to below. Such behavior is consistent with the view of suicidal behavior as a shift in control from value-based instrumental policies toward Pavlovian or merely unconditioned escape, whereby aversive internal states and cues trigger active withdrawal regardless of longer-term consequences (Dombrovski & Hallquist, 2022; Karvelis & Diaconescu, 2022).

As outlined in the introduction, normative studies of decisions under time cost show that reward-rate–maximizing policies can be implemented either by decision criteria that collapse as a trial unfolds or approximated by an urgency signal that ramps with elapsed time and amplifies the impact of current evidence (Drugowitsch et al., 2012; Tajima et al., 2016). These models account for premature quitting in general rather than specifically after losses. Converging animal and human data implicate lateral striatal circuitry in rapid loss-driven shifts in choice and show that executive, likely prefrontal, systems normally suppress these reflexive “lose-shift” tendencies, which become more prominent when cognitive control is taxed or underdeveloped (Ivan et al., 2018). The suicide attempters’ lose-quit behavior may thus be explained by a heightened or poorly regulated urgency process and/or a relative predominance of striatal stimulus-action responses over slower fronto-parietal temporal integration of reinforcement.

Heterogeneity analyses further refined this picture. Early-onset attempters showed the most pronounced deviation from controls, combining greater overpersistence with stronger loss-triggered immediate quitting. This pattern is consistent with the reactive, impulsive personality profile of individuals with early-onset versus late-onset suicidal behavior (Szanto et al., 2024; Szücs et al., 2020) and may mark a subtype in which crisis decisions are sensitive to short-term affective fluctuations. In contrast, stratifying attempters by medical lethality did not yield clear differences in overpersistence or loss-triggered immediate quitting, despite prior findings that high- and low-lethality attempters differ in exploration of options and adjustment to negative feedback (Tsypes et al., 2024). In this cohort, the age of first attempt may therefore be a more sensitive indicator of how re-engagement after losses is calibrated than the lethality of the most medically serious attempt, although these subgroup analyses are exploratory and should be interpreted with caution.

The tendency to persist too long once a wait is underway suggests a depression-related failure of dynamic re-evaluation. Unlike loss-triggered immediate quitting, which was most pronounced in attempters, poorer stopping calibration was not: overpersistence relative to controls reached significance in attempters and depressed non-suicidal participants, whereas ideators were intermediate and did not differ significantly from controls. In WTW, elapsed time carries information about the expected remaining delay and the opportunity cost of continued waiting, so quitting requires dynamically updating, based on accumulated experience, whether the expected reward still justifies the opportunity cost of waiting. This overpersistence was substantial rather than marginal: across all non-immediate-quit trials, participant-level mean excess waiting was roughly 2 to 3 s beyond the 3-s threshold (an excess comparable to the length of the optimal wait itself) and, because the task was time-limited, this excess waiting reduced the opportunities to begin a new, potentially rewarded trial. This pattern suggests difficulty adjusting stopping thresholds to the current time-cost structure rather than a simple preference for patience. This pattern complements findings from a high-persistence version of WTW, in which adults with major depression were less willing than controls to wait for delayed rewards when extended waiting maximized payoff, with no parallel alterations in a low-persistence variant (Mukherjee et al., 2020). Across the two studies, depression thus appears to disrupt the calibration of persistence in either direction (overpersistence when brief waits are optimal and underpersistence when long waits are optimal) rather than to reflect a fixed preference for patience or impatience. One possibility is that depression is associated with coarse or unstable estimates of when stopping is worthwhile, so persistence policies are only loosely tuned to environmental timing statistics. A different, not mutually exclusive, possibility is that depression alters how time and effort costs are valued. Indeed, in effort-based decision-making tasks, depressed people are less likely to choose high-effort, high-reward options and show reduced sensitivity to expected value (Treadway et al., 2012).

Viewed more broadly, WTW treats persistence not as a single tendency to continue but as a context-sensitive regulatory process, in which adaptive behavior requires sustaining engagement while persistence remains worthwhile, disengaging when expected returns no longer justify accumulating costs, and re-engaging when a setback does not eliminate the value of the next opportunity (Brandstätter & Bernecker, 2022; Wrosch et al., 2003). The same act of quitting can be adaptive or maladaptive depending on the temporal statistics of the environment (Lempert et al., 2018; 2023; McGuire & Kable, 2012, 2013, 2015), so the relevant capacity is calibration rather than persistence per se. Its repeated structure lets the WTW separate two decisions that a single persistence measure would conflate: whether an unrewarded outcome suppresses re-engagement with the next opportunity, and when an ongoing wait is terminated as costs accumulate. Applied to the present data, these decisions dissociated: reduced re-engagement after a setback was most pronounced in attempters, whereas excessive persistence once committed extended to depressed non-suicidal participants. The combination of both, seen in attempters, reflects a phase-specific miscalibration that a single overall index of persistence would obscure. WTW thus provides a controlled assay of responses to failure, disengagement, and re-engagement, though their generalization to suicidal crises remains to be established.

A central strength of the present study is therefore the use of a normative, process-resolved measure of persistence regulation. The WTW places participants in a time-pressured decision context with a running opportunity cost, allowing us to observe continuous re-evaluation of an ongoing choice and to dissociate starting from stopping decisions that are typically conflated in discounting, bandit, or reinforcement-timing tasks. The design further leverages a carefully characterized mid- to late-life cohort sampled into four comparison groups (attempters, ideators, depressed non-suicidal, and controls) and stratified by attempt lethality and age of first attempt, which strengthens inferences about specificity to suicidal behavior and permits targeted heterogeneity tests. Several limitations qualify these strengths. We examined a single condition in which brief persistence was optimal, so we do not know whether the same groups would miscalibrate persistence when long waits are rewarded or within an aversive rather than appetitive context. The cross-sectional design and mid- to late-life depression sample preclude conclusions about prospective risk and limit generalizability to younger populations, other diagnoses, attempts that unfold rapidly with little planning, and attempts occurring without reported suicidal ideation. Further, behavior was motivated by monetary reward in a laboratory context rather than by real-world relief from distress. Finally, we did not directly estimate latent constructs such as urgency, Pavlovian escape tendencies, or latent cause inference, which we invoked to interpret group differences. Future work should combine WTW variants that manipulate time-cost structure and outcome valence with computational modeling of these mechanisms, embed such tasks in longitudinal designs that track suicidal thoughts and behavior over time, and adapt similar paradigms for mobile or ecological momentary assessment-like settings where persistence and re-engagement can be studied closer to real suicidal crises.

In summary, attempters and depressed non-suicidal participants tended to persist beyond the payoff-maximizing point, failing to terminate an ongoing course of action as time costs accrued. Suicide attempters, however, were especially likely to quit immediately after a single unrewarded wait, foregoing a reward that could follow a 1–3 s wait. Thus, attempters differed from ideators more clearly in re-engagement after failure than in persistence once waiting had begun. This pattern fits with behaviorally framed accounts in which withdrawal from ambiguous but potentially rewarding contexts is negatively reinforced by short-term relief from anticipated disappointment or effort. To the extent that this bias extends to real life, it may contribute to overgeneralizing isolated setbacks as evidence that broader efforts are hopeless. Future work should test whether strategies that support re-engagement after failure reduce maladaptive disengagement in clinically relevant contexts.

## Supplementary Material

supplement

## Figures and Tables

**Table 1:** Demographic and clinical characteristics.

Variable	Attempters	Ideators	Depressed Non-Suicidal	Controls	*p*	*N*
*N*	87	59	63	68		277
Sex, female, *n* (%)	41 (47.1%)	26 (44.1%)	29 (46.0%)	41 (60.3%)	0.22	277
Race, White, *n* (%)	75 (86.2%)	49 (83.1%)	52 (82.5%)	60 (88.2%)	0.77	277
Married, *n* (%)	31 (35.6%)	17 (28.8%)	31 (49.2%)	41 (60.3%)	0.001	277
Age, years	62.1 (8.6)	62.7 (8.3)	64.3 (8.2)	65.6 (10.7)	0.09	277
Education, years	14.3 (3.1)	15.1 (2.5)	14.9 (2.4)	15.5 (2.7)	0.03	277
HRSD-16 at baseline	20.8 (5.9)	19.7 (5.6)	16.9 (3.8)	2.3 (2.1)	< 0.001	276
IQ (WTAR)	102.7 (15.7)	108.9 (14.4)	107.8 (14.4)	107.8 (12.1)	0.04	258
Cognitive control (EXIT25)	7.4 (3.5)	6.5 (3.6)	5.6 (3.2)	5.8 (3.3)	0.009	264
BIS Non-Planning	20.2 (8.2)	18.8 (7.5)	19.0 (7.7)	11.3 (5.1)	< 0.001	262
BIS Motor	12.9 (6.6)	12.7 (5.3)	12.1 (5.6)	9.3 (4.3)	< 0.001	262
BIS Attentional	15.2 (5.6)	14.4 (5.2)	13.9 (6.6)	9.1 (4.0)	< 0.001	262
BIS Total (mean item score)	1.6 (0.6)	1.5 (0.5)	1.5 (0.6)	1.0 (0.3)	< 0.001	262
NEO-FFI Neuroticism	39.6 (10.3)	40.8 (8.7)	34.7 (8.1)	21.4 (4.5)	< 0.001	238
NEO-FFI Extraversion	34.2 (8.9)	33.2 (6.3)	36.7 (7.5)	42.4 (6.2)	< 0.001	238
NEO-FFI Openness	36.3 (4.1)	37.0 (4.3)	37.1 (3.9)	37.9 (4.2)	0.20	238
NEO-FFI Agreeableness	45.7 (5.1)	44.2 (5.0)	45.8 (4.6)	47.3 (3.2)	0.008	238
NEO-FFI Conscientiousness	42.6 (8.7)	41.6 (7.9)	41.2 (8.2)	49.8 (4.6)	< 0.001	238
UPPS-P Negative Urgency	29.4 (7.6)	28.6 (7.3)	24.4 (6.4)	18.4 (4.2)	< 0.001	257
UPPS-P Positive Urgency	26.0 (9.0)	24.8 (8.3)	21.6 (6.9)	16.7 (4.1)	< 0.001	257
UPPS-P Lack of Premeditation	22.3 (6.9)	22.2 (5.9)	21.3 (5.3)	19.0 (4.1)	0.005	257
UPPS-P Lack of Perseverance	20.6 (5.7)	21.3 (5.4)	21.6 (5.3)	15.7 (3.3)	< 0.001	257

*Note*. Attempters = Suicide Attempters; Ideators = Suicide Ideators; Depressed = Depressed Non-Suicidal; Controls = Healthy Controls. Values are presented as *M (SD)* unless otherwise indicated. HRSD-16 = Hamilton Rating Scale for Depression, 17-item version scored without the suicide item. WTAR = Wechsler Test of Adult Reading; EXIT25 = Executive Interview (higher scores indicate worse executive functioning); BIS = Barratt Impulsiveness Scale; NEO-FFI = NEO Five-Factor Inventory; UPPS-P = UPPS-P Impulsive Behavior Scale. *p*-values are from one-way ANOVAs for continuous variables and chi-square tests for categorical variables.

**Table 2:** Mixed-effects models predicting quitting behavior.

Predictor	β	*SE*	*z*	*p*	Adj. β	Adj. *p*
***Panel A: Logistic Model for Immediate Quitting***						
Prior Trial Won (vs. Lost)	−4.08	0.18	−22.24	< 0.0001	−4.05	< 0.001
Group: Healthy Controls	−0.33	0.33	−0.98	0.33	−0.40	0.245
Group: Depressed Non-Suicidal	−0.65	0.35	−1.84	0.07	−0.85	0.020
Group: Suicide Ideators	−0.35	0.35	−1.00	0.32	−0.39	0.275
Prior Trial Won × Healthy Controls	0.96	0.24	4.04	< 0.0001	0.97	< 0.001
Prior Trial Won × Depressed Non-Suicidal	0.87	0.26	3.35	0.0008	0.96	< 0.001
Prior Trial Won × Suicide Ideators	0.97	0.26	3.80	0.0001	0.96	< 0.001
***Panel B: Cox Model for Quitting Latency***						
Group: Suicide Attempters	−0.68	0.29	−2.36	0.02	−0.74	0.010
Group: Depressed Non-Suicidal	−0.87	0.32	−2.73	0.006	−1.05	< 0.001
Group: Suicide Ideators	−0.54	0.33	−1.61	0.11	−0.70	0.030
Prior Trial Won (vs. Lost)	0.20	0.09	2.22	0.03	0.18	0.059
Prior Trial Won × Suicide Attempters	0.18	0.14	1.30	0.19	0.19	0.176
Prior Trial Won × Depressed Non-Suicidal	0.49	0.15	3.28	0.001	0.59	< 0.001
Prior Trial Won × Suicide Ideators	0.28	0.14	2.03	0.04	0.28	0.051

*Note*. Panel A: Reference group = Suicide Attempters. Panel B: Reference group = Healthy Controls. β = unstandardized coefficient; *SE* = standard error. Both models included random intercepts for participants. Prior Trial Won indicates whether the participant received the larger reward on the previous trial (coded 1) or quit before its arrival (coded 0). Adjusted β and adjusted *p*-values are from models that include age, education, WTAR-estimated IQ, and EXIT25 executive functioning as covariates. Unadjusted estimates are based on the full analytic samples: 277 participants and 9104 trials in Panel A and 274 participants and 9012 trials in Panel B. Adjusted estimates are based on participants with complete age, education, WTAR, and EXIT25 data: 257 participants and 8468 trials in Panel A and 254 participants and 8382 trials in Panel B. Complete-case adjusted models excluded 20 participants because of missing WTAR and/or EXIT25 data; age and education were complete for all participants. Twelve participants were missing both WTAR and EXIT25, seven were missing WTAR only, and one was missing EXIT25 only. The Cox analysis included 274 participants because three participants had no non-immediate-quit trial with an observable prior-trial outcome; their only eligible waiting trial was the first trial, for which prior outcome was undefined. Covariates were standardized before model estimation. In Panel B, negative coefficients indicate longer persistence (lower hazard of quitting). Because Prior Trial Won was coded 0 for a prior loss, group coefficients represent group differences following an unrewarded prior trial.

## Data Availability

De-identified data and analysis code are available on the Open Science Framework (https://osf.io/s89u4/).

## Appendix A. Supplementary data

Supplementary data to this article can be found online at https://doi.org/10.1016/j.brat.2026.105148.
