# Supplementary material for: When to quit: Calibration of voluntary persistence and attempted suicide in depression
Source: Behav Res Ther. Author manuscript; Available in PMC 2026 Sep 18. (PMC13583597; doi:10.1016/j.brat.2026.105148)
Supplement: Supplementary file 1 [file NIHMS2209761-supplement-supplement.docx]

**SUPPLEMENTAL MATERIALS**

***Supplemental Figure 1***

*Manipulation Checks*

*Note.* Survival curves display the probability of continued waiting over time, with waiting times censored at reward delivery. Immediate-quit trials (latency < 0.1 s) are excluded. The left panel shows that participants persisted longer following wins compared to losses, confirming sensitivity to reward outcomes. The right panel demonstrates that participants learned to quit faster over time when early rewards did not arrive, consistent with adaptive updating of timing expectations. Early and late trials were defined by a within-participant median split of trial number.

***Overview of Supplemental Tables***

The following supplemental tables present sensitivity and heterogeneity analyses examining the robustness of our main findings. Tables S1–S4 examine how persistence calibration varies by suicide attempt characteristics (lethality and age of onset). Tables S5–S6 confirm that results are robust to exclusion of participants who never moved the cursor. Tables S7–S8 present comprehensive sensitivity analyses testing whether individual difference variables (personality traits, cognitive functioning, impulsivity) account for the observed group differences. Tables S9–S16 present full models incorporating significant trait × prior trial outcome interactions alongside the primary group × prior trial outcome effects.

**Heterogeneity of Suicidal Behavior**

**Table S1.** Immediate Quitting: High vs. Low Attempt Lethality

**Table S2.** Quitting Latency: High vs. Low Attempt Lethality

**Table S3.** Immediate Quitting: Early vs. Late Onset Attempters

**Table S4.** Quitting Latency: Early vs. Late Onset Attempters

**Robustness Analyses**

**Table S5.** Immediate Quitting (Excluding n = 14 Cursor Non-Movers)

**Table S6.** Quitting Latency (Excluding n = 14 Cursor Non-Movers)

**Trait × Prior Trial Outcome Sensitivity Analyses**

**Table S7.** Trait Interactions for Persistence (Cox Models)

**Table S8.** Trait Interactions for Immediate Quits (Logistic Models)

**Full Models with Significant Trait Covariates**

**Table S9.** Quitting Latency with NEO-FFI Agreeableness

**Table S10.** Quitting Latency with BIS Non-Planning

**Table S11.** Quitting Latency with BIS Attentional

**Table S12.** Immediate Quitting with NEO-FFI Neuroticism

**Table S13.** Immediate Quitting with NEO-FFI Extraversion

**Table S14.** Immediate Quitting with NEO-FFI Agreeableness

**Table S15.** Immediate Quitting with NEO-FFI Conscientiousness

**Table S16.** Immediate Quitting with WTAR

***Table S1***

*Logistic Mixed-Effects Model Predicting Immediate Quitting: High vs. Low Attempt Lethality*

| **Predictor** | **β** | **SE** | **z** | **p** |
| --- | --- | --- | --- | --- |
| Prior Trial Won (vs. Lost) | -4.04 | 0.28 | -14.57 | < .0001 |
| Group: Low-Lethality Attempters | 0.001 | 0.47 | 0.002 | .998 |
| Group: Healthy Controls | -0.32 | 0.43 | -0.76 | .448 |
| Group: Depressed Non-Suicidal | -0.65 | 0.45 | -1.46 | .143 |
| Group: Suicide Ideators | -0.35 | 0.45 | -0.79 | .427 |
| Prior Trial Won × Low-Lethality | -0.07 | 0.37 | -0.18 | .858 |
| Prior Trial Won × Healthy Controls | 0.92 | 0.32 | 2.91 | .004 |
| Prior Trial Won × Depressed Non-Suicidal | 0.83 | 0.33 | 2.49 | .012 |
| Prior Trial Won × Suicide Ideators | 0.94 | 0.33 | 2.83 | .005 |

*Note.* Reference group = High-Lethality Attempters. β = unstandardized coefficient; SE = standard error.

***Table S2***

*Cox Mixed-Effects Model Predicting Quitting Latency: High vs. Low Attempt Lethality*

| **Predictor** | **β** | **SE** | **z** | **p** |
| --- | --- | --- | --- | --- |
| Group: High-Lethality Attempters | -0.64 | 0.37 | -1.74 | .083 |
| Group: Low-Lethality Attempters | -0.71 | 0.33 | -2.14 | .032 |
| Group: Depressed Non-Suicidal | -0.87 | 0.32 | -2.74 | .006 |
| Group: Suicide Ideators | -0.54 | 0.33 | -1.61 | .107 |
| Prior Trial Won (vs. Lost) | 0.20 | 0.09 | 2.22 | .026 |
| Prior Trial Won × High-Lethality | 0.17 | 0.18 | 0.97 | .331 |
| Prior Trial Won × Low-Lethality | 0.18 | 0.16 | 1.11 | .267 |
| Prior Trial Won × Depressed Non-Suicidal | 0.49 | 0.15 | 3.28 | .001 |
| Prior Trial Won × Suicide Ideators | 0.28 | 0.14 | 2.03 | .042 |

*Note.* Reference group = Healthy Controls. β = unstandardized coefficient; SE = standard error. Negative coefficients indicate longer persistence.

***Table S3***

*Logistic Mixed-Effects Model Predicting Immediate Quitting: Early vs. Late Onset Attempters*

| **Predictor** | **β** | **SE** | **z** | **p** |
| --- | --- | --- | --- | --- |
| Prior Trial Won (vs. Lost) | -5.35 | 0.40 | -13.29 | < .0001 |
| Group: Healthy Controls | -0.95 | 0.48 | -1.98 | .048 |
| Group: Late-Onset Attempters | -0.95 | 0.51 | -1.84 | .066 |
| Group: Depressed Non-Suicidal | -1.28 | 0.49 | -2.59 | .010 |
| Group: Suicide Ideators | -0.98 | 0.49 | -1.98 | .048 |
| Prior Trial Won × Healthy Controls | 2.22 | 0.43 | 5.19 | < .0001 |
| Prior Trial Won × Late-Onset | 1.90 | 0.45 | 4.21 | < .0001 |
| Prior Trial Won × Depressed Non-Suicidal | 2.13 | 0.44 | 4.83 | < .0001 |
| Prior Trial Won × Suicide Ideators | 2.23 | 0.44 | 5.10 | < .0001 |

*Note.* Reference group = Early-Onset Attempters. Early onset defined as first attempt before age 50. β = unstandardized coefficient; SE = standard error.

***Table S4***

*Cox Mixed-Effects Model Predicting Quitting Latency: Early vs. Late Onset Attempters*

| **Predictor** | **β** | **SE** | **z** | **p** |
| --- | --- | --- | --- | --- |
| Group: Early-Onset Attempters | -0.93 | 0.38 | -2.46 | .014 |
| Group: Late-Onset Attempters | -0.53 | 0.34 | -1.57 | .117 |
| Group: Depressed Non-Suicidal | -0.87 | 0.32 | -2.72 | .006 |
| Group: Suicide Ideators | -0.54 | 0.34 | -1.60 | .110 |
| Prior Trial Won (vs. Lost) | 0.20 | 0.09 | 2.23 | .026 |
| Prior Trial Won × Early-Onset | 0.40 | 0.19 | 2.09 | .037 |
| Prior Trial Won × Late-Onset | 0.06 | 0.16 | 0.36 | .719 |
| Prior Trial Won × Depressed Non-Suicidal | 0.49 | 0.15 | 3.28 | .001 |
| Prior Trial Won × Suicide Ideators | 0.28 | 0.14 | 2.03 | .042 |

*Note.* Reference group = Healthy Controls. Early onset defined as first attempt before age 50. β = unstandardized coefficient; SE = standard error. Negative coefficients indicate longer persistence.

***Table S5***

*Logistic Mixed-Effects Model Predicting Immediate Quitting (Excluding n = 14 Participants Who Never Moved Cursor)*

| **Predictor** | **β** | **SE** | **z** | **p** |
| --- | --- | --- | --- | --- |
| (Intercept) | 0.79 | 0.24 | 3.31 | .0009 |
| Group: Healthy Controls | -0.27 | 0.34 | -0.80 | .427 |
| Group: Depressed Non-Suicidal | -0.64 | 0.36 | -1.78 | .075 |
| Group: Suicide Ideators | -0.32 | 0.36 | -0.88 | .379 |
| Prior Trial Won (vs. Lost) | -4.08 | 0.18 | -22.12 | < .0001 |
| Prior Trial Won × Healthy Controls | 0.95 | 0.24 | 4.00 | < .0001 |
| Prior Trial Won × Depressed Non-Suicidal | 0.86 | 0.26 | 3.32 | .0009 |
| Prior Trial Won × Suicide Ideators | 0.97 | 0.26 | 3.78 | .0002 |

*Note.* Reference group = Suicide Attempters. β = unstandardized coefficient; SE = standard error.

***Table S6***

*Cox Mixed-Effects Model Predicting Quitting Latency (Excluding n = 14 Participants Who Never Moved Cursor)*

| **Predictor** | **β** | **SE** | **z** | **p** |
| --- | --- | --- | --- | --- |
| Group: Suicide Attempters | -0.66 | 0.29 | -2.27 | .023 |
| Group: Depressed Non-Suicidal | -0.86 | 0.32 | -2.69 | .007 |
| Group: Suicide Ideators | -0.55 | 0.34 | -1.61 | .107 |
| Prior Trial Won (vs. Lost) | 0.20 | 0.09 | 2.21 | .027 |
| Prior Trial Won × Suicide Attempters | 0.19 | 0.14 | 1.37 | .170 |
| Prior Trial Won × Depressed Non-Suicidal | 0.49 | 0.15 | 3.29 | .001 |
| Prior Trial Won × Suicide Ideators | 0.28 | 0.14 | 2.00 | .045 |

*Note.* Reference group = Healthy Controls. β = unstandardized coefficient; SE = standard error. Negative coefficients indicate longer persistence.

***Table S7***

*Sensitivity Analyses: Trait × Prior Trial Outcome Interactions for Persistence (Cox Models)*

| **Variable** | **Int. β** | **SE** | **z** | **p** |
| --- | --- | --- | --- | --- |
| UPPS-P Negative Urgency | 0.11 | 0.06 | 1.89 | .058 |
| UPPS-P Positive Urgency | 0.01 | 0.05 | 0.10 | .917 |
| UPPS-P Lack of Premeditation | 0.09 | 0.06 | 1.58 | .115 |
| UPPS-P Lack of Perseverance | -0.03 | 0.06 | -0.53 | .593 |
| NEO-FFI Neuroticism | 0.02 | 0.05 | 0.33 | .739 |
| NEO-FFI Extraversion | -0.06 | 0.06 | -1.01 | .313 |
| NEO-FFI Openness | -0.03 | 0.06 | -0.55 | .584 |
| **NEO-FFI Agreeableness** | **-0.12** | **0.06** | **-2.21** | **.027** |
| NEO-FFI Conscientiousness | -0.03 | 0.06 | -0.57 | .571 |
| EXIT25 (Executive Function) | -0.03 | 0.05 | -0.67 | .502 |
| WTAR (IQ Estimate) | 0.03 | 0.05 | 0.48 | .628 |
| **BIS Non-Planning** | **0.11** | **0.06** | **2.02** | **.043** |
| BIS Motor | 0.08 | 0.06 | 1.44 | .150 |
| **BIS Attentional** | **-0.13** | **0.06** | **-2.11** | **.035** |
| BIS Total (mean item score) | 0.05 | 0.06 | 0.90 | .368 |

*Note.* All trait variables are standardized. Model: coxme(Surv(latency, quit) ~ Prior Trial Won × scale(TRAIT) + (1|ID)). Int. = Trait × Prior Trial Won interaction. β = unstandardized coefficient; SE = standard error. WTAR = Wechsler Test of Adult Reading; EXIT25 = Executive Interview (higher scores indicate worse executive function); BIS = Barratt Impulsiveness Scale; NEO-FFI = NEO Five-Factor Inventory; UPPS-P = UPPS-P Impulsive Behavior Scale.

***Table S8***

*Sensitivity Analyses: Trait × Prior Trial Outcome Interactions for Immediate Quits (Logistic Models)*

| **Variable** | **Int. β** | **SE** | **z** | **p** |
| --- | --- | --- | --- | --- |
| UPPS-P Negative Urgency | 0.03 | 0.10 | 0.33 | .743 |
| UPPS-P Positive Urgency | 0.09 | 0.09 | 1.01 | .313 |
| UPPS-P Lack of Premeditation | -0.06 | 0.09 | -0.58 | .560 |
| UPPS-P Lack of Perseverance | -0.01 | 0.09 | -0.06 | .951 |
| **NEO-FFI Neuroticism** | **-0.44** | **0.10** | **-4.44** | **< .0001** |
| **NEO-FFI Extraversion** | **0.44** | **0.10** | **4.57** | **< .0001** |
| NEO-FFI Openness | -0.12 | 0.10 | -1.27 | .204 |
| **NEO-FFI Agreeableness** | **-0.20** | **0.09** | **-2.15** | **.031** |
| **NEO-FFI Conscientiousness** | **0.36** | **0.10** | **3.55** | **.0004** |
| EXIT25 (Executive Function) | 0.01 | 0.08 | 0.10 | .919 |
| **WTAR (IQ Estimate)** | **0.30** | **0.09** | **3.32** | **.0009** |
| BIS Non-Planning | -0.03 | 0.09 | -0.38 | .707 |
| BIS Motor | 0.01 | 0.09 | 0.06 | .953 |
| BIS Attentional | 0.09 | 0.09 | 1.02 | .310 |
| BIS Total (mean item score) | 0.02 | 0.09 | 0.19 | .848 |

*Note.* All trait variables are standardized. Model: glmer(immQuit ~ Prior Trial Won × scale(TRAIT) + (1|ID), family = binomial). Int. = Trait × Prior Trial Won interaction. β = unstandardized coefficient; SE = standard error. WTAR = Wechsler Test of Adult Reading; EXIT25 = Executive Interview (higher scores indicate worse executive function); BIS = Barratt Impulsiveness Scale; NEO-FFI = NEO Five-Factor Inventory; UPPS-P = UPPS-P Impulsive Behavior Scale.

***Table S9***

*Cox Model Predicting Quitting Latency: Group × Prior Trial Outcome + NEO-FFI Agreeableness × Prior Trial Outcome*

| **Predictor** | **β** | **SE** | **z** | **p** |
| --- | --- | --- | --- | --- |
| Group: Suicide Attempters | -0.57 | 0.32 | -1.77 | .077 |
| Group: Depressed Non-Suicidal | -0.79 | 0.36 | -2.19 | .028 |
| Group: Suicide Ideators | -0.35 | 0.38 | -0.93 | .354 |
| Prior Trial Won (vs. Lost) | 0.36 | 0.11 | 3.29 | .001 |
| NEO-FFI Agreeableness | 0.09 | 0.12 | 0.71 | .475 |
| Prior Trial Won × Suicide Attempters | 0.11 | 0.16 | 0.68 | .495 |
| Prior Trial Won × Depressed Non-Suicidal | 0.27 | 0.18 | 1.55 | .120 |
| Prior Trial Won × Suicide Ideators | 0.11 | 0.16 | 0.69 | .493 |
| Prior Trial Won × Agreeableness | -0.11 | 0.06 | -1.82 | .068 |

*Note.* Reference group = Healthy Controls. β = unstandardized coefficient; SE = standard error. NEO-FFI = NEO Five-Factor Inventory.

***Table S10***

*Cox Model Predicting Quitting Latency: Group × Prior Trial Outcome + BIS Non-Planning × Prior Trial Outcome*

| **Predictor** | **β** | **SE** | **z** | **p** |
| --- | --- | --- | --- | --- |
| Group: Suicide Attempters | -0.57 | 0.31 | -1.81 | .070 |
| Group: Depressed Non-Suicidal | -0.76 | 0.33 | -2.27 | .023 |
| Group: Suicide Ideators | -0.45 | 0.35 | -1.30 | .195 |
| Prior Trial Won (vs. Lost) | 0.17 | 0.10 | 1.67 | .095 |
| BIS Non-Planning | -0.10 | 0.12 | -0.88 | .380 |
| Prior Trial Won × Suicide Attempters | 0.17 | 0.16 | 1.09 | .275 |
| Prior Trial Won × Depressed Non-Suicidal | 0.52 | 0.17 | 3.12 | .002 |
| Prior Trial Won × Suicide Ideators | 0.36 | 0.15 | 2.41 | .016 |
| Prior Trial Won × BIS Non-Planning | 0.06 | 0.06 | 0.97 | .333 |

*Note.* Reference group = Healthy Controls. β = unstandardized coefficient; SE = standard error. BIS = Barratt Impulsiveness Scale.

***Table S11***

*Cox Model Predicting Quitting Latency: Group × Prior Trial Outcome + BIS Attentional × Prior Trial Outcome*

| **Predictor** | **β** | **SE** | **z** | **p** |
| --- | --- | --- | --- | --- |
| Group: Suicide Attempters | -0.88 | 0.31 | -2.87 | .004 |
| Group: Depressed Non-Suicidal | -0.97 | 0.33 | -2.99 | .003 |
| Group: Suicide Ideators | -0.67 | 0.35 | -1.93 | .053 |
| Prior Trial Won (vs. Lost) | 0.00 | 0.10 | -0.02 | .986 |
| BIS Attentional | 0.18 | 0.11 | 1.61 | .107 |
| Prior Trial Won × Suicide Attempters | 0.47 | 0.15 | 3.02 | .003 |
| Prior Trial Won × Depressed Non-Suicidal | 0.70 | 0.16 | 4.28 | < .0001 |
| Prior Trial Won × Suicide Ideators | 0.55 | 0.15 | 3.62 | .0003 |
| Prior Trial Won × BIS Attentional | -0.24 | 0.07 | -3.47 | .0005 |

*Note.* Reference group = Healthy Controls. β = unstandardized coefficient; SE = standard error. BIS = Barratt Impulsiveness Scale.

***Table S12***

*Logistic Model Predicting Immediate Quitting: Group × Prior Trial Outcome + NEO-FFI Neuroticism × Prior Trial Outcome*

| **Predictor** | **β** | **SE** | **z** | **p** |
| --- | --- | --- | --- | --- |
| Group: Healthy Controls | 0.16 | 0.49 | 0.33 | .743 |
| Group: Depressed Non-Suicidal | -0.38 | 0.41 | -0.95 | .345 |
| Group: Suicide Ideators | -0.40 | 0.39 | -1.03 | .303 |
| Prior Trial Won (vs. Lost) | -4.30 | 0.21 | -20.25 | < .0001 |
| NEO-FFI Neuroticism | 0.49 | 0.19 | 2.56 | .010 |
| Prior Trial Won × Healthy Controls | 0.92 | 0.34 | 2.73 | .006 |
| Prior Trial Won × Depressed Non-Suicidal | 0.45 | 0.31 | 1.43 | .152 |
| Prior Trial Won × Suicide Ideators | 1.11 | 0.29 | 3.88 | .0001 |
| Prior Trial Won × Neuroticism | -0.33 | 0.14 | -2.38 | .017 |

*Note.* Reference group = Suicide Attempters. β = unstandardized coefficient; SE = standard error. NEO-FFI = NEO Five-Factor Inventory.

***Table S13***

*Logistic Model Predicting Immediate Quitting: Group × Prior Trial Outcome + NEO-FFI Extraversion × Prior Trial Outcome*

| **Predictor** | **β** | **SE** | **z** | **p** |
| --- | --- | --- | --- | --- |
| Group: Healthy Controls | -0.26 | 0.41 | -0.64 | .525 |
| Group: Depressed Non-Suicidal | -0.48 | 0.40 | -1.21 | .226 |
| Group: Suicide Ideators | -0.44 | 0.39 | -1.12 | .264 |
| Prior Trial Won (vs. Lost) | -4.37 | 0.21 | -20.93 | < .0001 |
| NEO-FFI Extraversion | -0.42 | 0.15 | -2.77 | .006 |
| Prior Trial Won × Healthy Controls | 1.11 | 0.28 | 3.94 | < .0001 |
| Prior Trial Won × Depressed Non-Suicidal | 0.46 | 0.31 | 1.49 | .136 |
| Prior Trial Won × Suicide Ideators | 1.16 | 0.29 | 4.03 | < .0001 |
| Prior Trial Won × Extraversion | 0.38 | 0.11 | 3.53 | .0004 |

*Note.* Reference group = Suicide Attempters. β = unstandardized coefficient; SE = standard error. NEO-FFI = NEO Five-Factor Inventory.

***Table S14***

*Logistic Model Predicting Immediate Quitting: Group × Prior Trial Outcome + NEO-FFI Agreeableness × Prior Trial Outcome*

| **Predictor** | **β** | **SE** | **z** | **p** |
| --- | --- | --- | --- | --- |
| Group: Healthy Controls | -0.70 | 0.39 | -1.82 | .069 |
| Group: Depressed Non-Suicidal | -0.52 | 0.40 | -1.31 | .189 |
| Group: Suicide Ideators | -0.26 | 0.39 | -0.67 | .506 |
| Prior Trial Won (vs. Lost) | -4.41 | 0.21 | -21.31 | < .0001 |
| NEO-FFI Agreeableness | 0.21 | 0.14 | 1.48 | .139 |
| Prior Trial Won × Healthy Controls | 1.54 | 0.27 | 5.73 | < .0001 |
| Prior Trial Won × Depressed Non-Suicidal | 0.51 | 0.31 | 1.65 | .099 |
| Prior Trial Won × Suicide Ideators | 0.97 | 0.29 | 3.41 | .001 |
| Prior Trial Won × Agreeableness | -0.29 | 0.10 | -2.90 | .004 |

*Note.* Reference group = Suicide Attempters. β = unstandardized coefficient; SE = standard error. NEO-FFI = NEO Five-Factor Inventory.

***Table S15***

*Logistic Model Predicting Immediate Quitting: Group × Prior Trial Outcome + NEO-FFI Conscientiousness × Prior Trial Outcome*

| **Predictor** | **β** | **SE** | **z** | **p** |
| --- | --- | --- | --- | --- |
| Group: Healthy Controls | -0.62 | 0.41 | -1.53 | .126 |
| Group: Depressed Non-Suicidal | -0.57 | 0.40 | -1.42 | .155 |
| Group: Suicide Ideators | -0.33 | 0.39 | -0.85 | .397 |
| Prior Trial Won (vs. Lost) | -4.41 | 0.21 | -21.32 | < .0001 |
| NEO-FFI Conscientiousness | 0.00 | 0.15 | 0.01 | .995 |
| Prior Trial Won × Healthy Controls | 1.22 | 0.28 | 4.35 | < .0001 |
| Prior Trial Won × Depressed Non-Suicidal | 0.60 | 0.31 | 1.97 | .049 |
| Prior Trial Won × Suicide Ideators | 1.09 | 0.28 | 3.85 | .0001 |
| Prior Trial Won × Conscientiousness | 0.25 | 0.11 | 2.16 | .031 |

*Note.* Reference group = Suicide Attempters. β = unstandardized coefficient; SE = standard error. NEO-FFI = NEO Five-Factor Inventory.

***Table S16***

*Logistic Model Predicting Immediate Quitting: Group × Prior Trial Outcome + WTAR × Prior Trial Outcome*

| **Predictor** | **β** | **SE** | **z** | **p** |
| --- | --- | --- | --- | --- |
| Group: Healthy Controls | -0.36 | 0.34 | -1.06 | .290 |
| Group: Depressed Non-Suicidal | -0.77 | 0.36 | -2.14 | .032 |
| Group: Suicide Ideators | -0.36 | 0.35 | -1.03 | .304 |
| Prior Trial Won (vs. Lost) | -3.99 | 0.19 | -21.28 | < .0001 |
| WTAR Standard Score | -0.36 | 0.13 | -2.79 | .005 |
| Prior Trial Won × Healthy Controls | 0.88 | 0.24 | 3.60 | .0003 |
| Prior Trial Won × Depressed Non-Suicidal | 0.87 | 0.27 | 3.22 | .001 |
| Prior Trial Won × Suicide Ideators | 0.85 | 0.26 | 3.27 | .001 |
| Prior Trial Won × WTAR | 0.23 | 0.09 | 2.45 | .014 |

*Note.* Reference group = Suicide Attempters. β = unstandardized coefficient; SE = standard error. WTAR = Wechsler Test of Adult Reading.
